# A Numerical Model to Predict the Relaxation Phenomena in Thermoset Polymers and Their Effects on Residual Stress during Curing—Part I: A Theoretical Formulation and Numerical Evaluation of Relaxation Phenomena

**DOI:** 10.3390/polym16101433

**Published:** 2024-05-18

**Authors:** Raffaele Verde, Alberto D’Amore, Luigi Grassia

**Affiliations:** Department of Engineering, University of Campania “Luigi Vanvitelli”, via Roma, 29, 81031 Aversa, Italy; raffaele.verde1@unicampania.it (R.V.); alberto.damore@unicampania.it (A.D.)

**Keywords:** epoxy, viscoelasticity, cure, structural relaxation, numerical simulation

## Abstract

This paper analyzes the effect of crosslinking reactions on a thermoset polymer’s viscoelastic properties. In particular, a numerical model to predict the evolution of epoxy’s mechanical properties during the curing process is proposed and implemented in an Ansys APDL environment. A linear viscoelastic behavior is assumed, and the scaling of viscoelastic properties in terms of the temperature and degree of conversion is modeled using a modified version of the TNM (Tool–Narayanaswamy–Mohynian) model. The effects of the degree of conversion and structural relaxation on epoxy’s relaxation times are simultaneously examined for the first time. This formulation is based on the thermo-rheological and chemo-rheological simplicities hypothesis and can predict the evolution of epoxy’s relaxation phenomena. The thermal–kinetic reactions of curing are implemented in a homemade routine written in APDL language, and the structural module of Ansys is used to predict the polymer’s creep and stress relaxation curves at different temperatures and degrees of conversion.

## 1. Introduction

During their manufacturing, thermosetting polymers and their composites are generally put under a vacuum, and thermal loads are applied to induce the resin’s crosslinking reaction. During this process, chemical reactions transform epoxy from a liquid-like to an almost solid-like material [1]; many phenomena of different natures take part, and most act simultaneously and are reciprocally linked. The most important ones are the thermal expansion/contraction due to positive/negative temperature variation, the chemical crosslinking reaction, the viscoelasticity, and the structural relaxation [2].

Structural relaxation is an unavoidable consequence of the glass transition. Epoxy’s crosslinked molecular structures are amorphous (because of the randomly ordered structure), and when it is cooled from its liquid to glassy state, the glass transition occurs. The material is metastable, so its structure and its structure-sensitive properties try to evolve toward an equilibrium. This physical phenomenon is called structural relaxation, and the evolution of the glassy structure during structural relaxation can be known following the evolution of structure-sensitive properties (like the volume, the enthalpy, and the entropy) through differential thermal analysis techniques. The kinetics of structural relaxation have been studied for a long time, but it remains an open question of polymer physics [3].

Different theories and models were proposed to understand the glass transition and its kinetics. The two first and most important models to describe the evolution of structure and structure-sensitive properties during the glass transition are the KAHR [4] and the TNN [5] models. They are developed independently but have similar physical meanings. In the KAHR model, the glassy state is measured by the departure from the equilibrium; in the TNM model, instead, the glassy state is identified by introducing the concept of fictive temperature. Based on these models, many other studies were developed to model and explain all the main characteristics of that phenomenon: the memory, the non-exponentiality, and the non-linearity [6,7,8,9,10].

Non-exponentiality implies that structural relaxation is subdivided into several processes, each of which has its own relaxation time. Non-linearity signifies that the structural relaxation response depends on the applied load’s direction and magnitude. The memory implies that the relaxation phenomena depend not only on the applied perturbation but also on the component’s previous thermal history.

Like almost all polymers, epoxy is a viscoelastic material. In addition, it demonstrates a behavior intermediately between an elastic solid and a viscous fluid, so its mechanical properties are time-dependent, and it is affected by creep and stress relaxation phenomena [11,12]. Many efforts were made to model the polymer’s viscoelasticity mathematically [13,14,15]: the viscoelastic functions generally depend on temperature and applied stress for polymers and other isotropic and homogeneous materials; they are interconnected, and their relative placement in the time domain was described by Grassia et al. [16]

A linear behavior is assumed for the material under study: the relaxation functions do not depend on the mechanical input applied to the system. Moreover, the effect of temperature on relaxation phenomena is only used to translate the viscoelastic functions on their timescale, the hypothesis of thermo-rheological simplicity [17].

During curing, the material changes its chemical structure, so its mechanical properties evolve as a reaction progress function [18]. Moreover, the curing process of epoxies is exothermic. Therefore, internal heat generation inevitably affects the thermal field and the temperature-activated reaction kinetics, so the thermal and kinetics problems are mutually linked.

The effect of structural relaxation on the viscoelastic functions was treated in many studies [19,20] as the effect of curing on viscoelasticity [21,22,23], but these three phenomena have never been discussed simultaneously. In this work, an attempt to model the interconnections of all these phenomena is made, in order to predict the kinetics of epoxy relaxation phenomena and evaluate their effects on polymeric materials’ relaxation properties (in part I of this work) and residual stresses arising during curing (in part II of this work).

Since curing causes a molecular structure change, the first step is to model how the degree of crosslinking affects the mechanical properties. Crosslinking creates a three-dimensional network of bonds between polymer chains, increasing the material’s strength and stiffness. In addition, the curing increases the glass temperature and the viscoelastic relaxation time. Various models were used to understand how the curing process affects epoxy’s mechanical behavior. In many of them [24,25,26,27], an elastic behavior was supposed to be exhibited, and a dependence of elastic modulus on temperature and the degree of conversion was assumed. They are simplified models, and, in some cases, the results are in good accordance with experimental ones, but they cannot consider stress relaxation due to the viscoelastic nature of polymers.

Kim and White proposed a viscoelastic model [21]. They experimentally evaluated the effect of curing on the epoxy’s relaxation time, proposing a viscoelastic formulation that can be considered the “gold standard” and was often adopted as a benchmark to evaluate simplified models [17]. In particular, they conducted experiments by curing epoxy coupons until reaching different conversion degrees. Then, the corresponding viscoelastic properties were evaluated at different temperatures for each degree of curing, and an expression for the time–temperature–degree of the conversion shift factor was proposed.

On the basis of Kim and White’s experimental data, this paper proposes a new expression to model the simultaneous effects of curing and structural relaxation on viscoelastic relaxation times to predict the kinetics of epoxy relaxation phenomena. In the second part of this work, the theoretical model proposed in this article will be used to evaluate the effect of the curing process on the residual stresses in a polymeric part subjected to an arbitrary thermal history.

The leading causes of internal stresses, in that case, are to be searched in the non-uniform thermal and viscoelastic properties due to the different thermal histories that each point of the curing volume suffers during the process and the chemical shrinkage associated with the 3D crosslinking formation.

In addition, since the glass transition is a kinetic transformation, if a polymeric component is subjected to non-uniform thermal history during its cooling from a liquid to glassy state, at the end of cooling, different points have at the same temperature different specific volumes, and for the equilibrium of adjacent areas, residual stresses arise, as shown by D’Amore et al. [20], who numerically estimated structural relaxation-induced stresses in a polystyrene part, showing that the structural relaxation phenomena play a significant role in the development of the internal tensional state.

## 2. Materials and Methods

### 2.1. Mathematical Formulation

The main parameter that identifies the state of the curing process is the degree of conversion, defined as the following:

(1) $$\alpha =\frac{H(t)}{H_{r}}$$

where *H*(*t*) is the internal heat produced at time t by the curing reaction, and *H_r_* is the total heat produced at the end of the reaction.

The rate of conversion is a function of temperature and time. In the framework of this modeling approach, kinetic equations are taken from the work of Bogetti et al. [28]. It is a well-validated model for a high-Tg epoxy for aeronautical applications:

(2) $$\frac{d\alpha }{dt}=\left(k_{1}+k_{2}\;\alpha \right)\left(0.47-\alpha \right)\;\;\;\;\;\;\;\;\;for\;\left(\alpha \leq \;0.3\right)$$

(3) $$\frac{d\alpha }{dt}=k_{3}\left(1-\alpha \right)\;\;\;\;\;\;\;\;for\;(\alpha >0.3)$$

*k*_1_, *k*_2_, and *k*_3_ follow the Arrhenius law:

(4) $$k_{i}=A_{i}\;Exp\left(-\frac{{∆E}_{i}}{RT}\right)\;\;\;\;\;i=1,2,3\;\;\;\;\;\;$$

where $k_{i\;}$is a material coefficient, *T* is the absolute temperature, *R* is the gas universal constant, and ${∆E}_{i}$ are the activation energies reported in Table 1.

The curing is an exothermic process, and the internal heat is assumed to be proportional to the rate of conversion:

(5) $$Q=\rho \;H_{r}\frac{\partial \alpha }{\partial t}\;\;\;\;\;\;\;$$

where *H_r_* is the heat generated per unit mass, and α is the degree of conversion, expressing the mass reacted to the total mass ratio. The reaction rate is a temperature-activated phenomenon, and thus is influenced by the exothermic process. Consequently, the thermal profiles and the cure kinetics are solved coupled.

In the framework of this article, isothermal configurations are analyzed, so internal heat generation is neglected in this case. In part II of this work, a non-isothermal geometry is considered; in that case, the effects of the exothermic reaction on the thermal fields are relevant.

The constitutive equation for linear viscoelasticity is adopted in this case, and the stress–strain formula can be expressed as the following:

(6) $$\sigma_{ij}=\int_{0}^{\xi }G(\alpha ,\xi -\xi^{\prime })\frac{de_{ij}}{d\xi^{\prime }}\;\;{d\xi }^{\prime }+\delta_{ij}\int_{0}^{\xi }\frac{1}{3}\;K(\alpha ,\xi -\xi^{\prime })\frac{d(\varepsilon -\varepsilon_{free})}{d\xi^{\prime }}\;\;{d\xi }^{\prime }\;\;$$

where $\varepsilon_{free}\;$ is the free strain due to the non-mechanical loads. In the case under study, it is the sum of the thermal and chemical strain. The chemical strain considers the contraction due to crosslinking network formation.

*G* e *K* are the shear and the bulk relaxation moduli. They are strong functions of temperature and the degree of curing and can be expressed by the Prony series:

(7) $$G\left(\xi \right)=G_{0\;}\left[\;\alpha_{\infty }^{G}+\sum_{i=1}^{n_{G}}\alpha_{i}^{G}\exp\left(-\frac{\xi }{\tau_{i}^{G}}\;\right)\right]\;\;\;\;$$

(8) $$K\left(\xi \right)=K_{0}\;\left[\;\alpha_{\infty }^{K}+\sum_{i=1}^{n_{K}}\alpha_{i}^{K}\exp\left(-\frac{\xi }{\tau_{i}^{K}}\right)\right]$$

$\alpha_{\infty }^{G}$ and $\alpha_{\infty }^{K}$ are defined as follows:

(9) $$\alpha_{\infty }^{G}=\frac{G_{0}-G_{\infty }}{G_{0}}$$

(10) $$\alpha_{\infty }^{K}=\frac{K_{0}-K_{\infty }}{K_{0}}$$

*G*_0_ e *K*_0_ are the unrelaxed shear and bulk moduli (glassy moduli), and $G_{\infty }$ e $K_{\infty }$ are the fully relaxed shear and bulk moduli (rubbery moduli). It is assumed that the shear rubbery moduli are independent of the degree of conversion, and its value is near zero [21].

The bulk modulus is assumed to be constant in time. Its relaxation can be neglected, varying only by a factor of 2 ÷ 3 along the timescale, and the difference between the epoxy’s unrelaxed and relaxed bulk modulus is very small [29].

The shear relaxation modulus, numerically, is expressed as a series of Maxwell models, each of them with a characteristic time, $\tau_{i}^{G}$, and associated weight factor, $\alpha_{i}^{G}$.

ξ is the reduced time and can be expressed as the following:

(11) $$\xi =\int_{0}^{t}\frac{1}{a(T,\alpha )}\;dt^{\prime }\;\;\;\;\;$$

a(T,α) is the temperature–degree of conversion shift factor.

Since the epoxy structure changes during the curing, the relaxation times and the viscoelastic properties are functions of the degree of conversion. The effects of curing on viscoelastic properties are considered in the shift factor’s expression, a(T,α).

Our approach adopts the following hypotheses, already well validated for a large class of polymeric materials [30,31,32,33]:

(a) Thermo-rheological simplicity: At a given degree of curing, the mechanical response at short times and high temperatures is the same as the response at low temperatures and long times. It is a well-validated hypothesis for a large class of amorphous polymers, including epoxy, for a wide temperature range;
(b) Chemo-rheological simplicity: The shape of the viscoelastic curve is the same at different degrees of conversion. The only effect of the degree of conversion is to translate the curves on the timescale. The hypothesis of time–degree of the cure superposition was introduced by Adolf and Martin [30], and its validity has been a debated issue among many researchers. In particular, Simon et al. [31] proposed a mathematical formulation based on TCS to describe the cure-dependent viscoelasticity of epoxy after gelation, showing that TCS is probably not strictly valid but represents a good approximation for engineering purposes. Similarly, Saseendran et al. [33] reported that the epoxy’s viscoelastic behavior, at any degree of conversion, can be obtained from a single master curve by scaling it along the timescale using an appropriate shift factor.

The relaxation times, *τ*, at a generic temperature and degree of conversion, can be expressed as the following:

(12) $$\;\tau \left(T,\alpha \right)=\tau_{R}/a\left(T,\alpha \right)\;$$

The reference state is assumed to be the glass transition temperature, *T_g_*, at each degree of conversion. The glass transition temperature generally evolves as a quadratic function of the degree of conversion [21].

(13) $$T_{g}\left(\alpha \right)=b_{1}+b_{2}\;\alpha +b_{3}\;\alpha^{2}\;$$

where *b*_1_, *b*_2_, and *b*_3_ are material constants and are reported in Equation (29).

It is hypothesized that, independently of the degree of the conversion, the relaxation time does not change at the glass transition temperature.

At a given degree of conversion, the structural relaxation shift factor is defined through the following modified TNM [5] equation:

(14) $$a\left(T,\alpha ,T_{f}\right)=exp\left(-\frac{\Delta H\left(\alpha \right)}{R}\;\left(\;\frac{x\left(\alpha \right)}{T}+\frac{1-x\left(\alpha \right)}{T_{f}}-\frac{1}{T_{g}(\alpha )}\;\right)\right)\;$$

(15) $$\Delta H\left(\alpha \right)=k_{4}+k_{5}\alpha +k_{6}\alpha^{2}\;$$

(16) $$x\left(\alpha \right)=k_{7}+k_{8}\alpha \;$$

**Figure 1:**
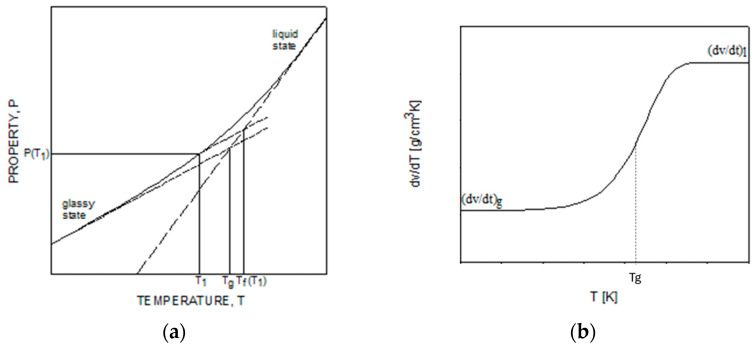
Definition of fictive temperature. (**a**) Schematic of a generic property P vs. temperature during the glass transition (**b**) Temperature derivative of volume during the glass transition.

Δ*H* is the activation energy and *x* is the non-linearity parameter that allows us to distinguish the effect of temperature and the structure on the relaxation time. Both parameters are functions of the degree of conversion, and *k*_4_, *k*_5_, *k*_6_, *k*_7_, and *k*_8_ are material constants and are reported in Equations (31) and (32). *T_f_* is the fictive temperature. The concept of fictive temperature was introduced for the first time by Tool [34] to univocally identify the glassy state. It can be obtained by assuming a linear behavior of the thermodynamic properties deep in the glassy state and in the equilibrium region, as shown schematically in Figure 1a.

The evolution of a generic property p in the glassy state can be calculated using the following equation:

(17) $$\frac{dp}{dT}=\alpha_{pg}\left(T\right)+\left(\alpha_{pg}\left(T\right)-\alpha_{pl}\left(T\right)\right)\;\;\frac{dT_{f}}{dT}$$

where $\alpha_{pg}$ and $\alpha_{pl}$ are the coefficients of properties in the glassy and liquid states, respectively. In Figure 1b, the temperature derivative of the volume is reported. In that case, $\alpha_{pg}$ and $\alpha_{pl}$ are the coefficients of thermal expansion deep in the glassy state and the equilibrium region.

The first term of Equation (17) gives the instantaneous change in the properties, while the second describes the relaxation of properties towards the equilibrium value.

The fictive temperature’s evolution in time can be evaluated according to the TNM model [5]:

(18) $$T_{f}\left(t\right)=T\left(t\right)-\int_{0}^{t}M(\xi \left(t\right)-\xi \left(t^{\prime }\right))\;\;\frac{dT(t^{\prime })}{dt^{\prime }}dt^{\prime }\;$$

where $\xi \left(t\right)$ is the reduced time. Following Equation (14), it can be expressed as the following:

(19) $$\xi \left(t\right)=\int_{0}^{t^{\prime }}exp\left[\frac{\Delta H\left(\alpha \right)}{R}\;\left(\;\frac{x\left(\alpha \right)}{T}+\frac{1-x\left(a\right)}{T_{f}}-\frac{1}{T_{g}(\alpha )}\;\right)\right]dt^{\prime }\;$$

An important characteristic of the glass relaxation phenomenon is that the relaxation rate depends on time, temperature, and the temperature history (memory effect).

M(ξ) is the memory function and can be expressed as a stretched exponential function.

(20) $$M\left(\xi \right)=exp[-{\left(\frac{\xi }{\tau_{M}}\right)}^{\beta_{M}}]$$

where β_M_ is the shape parameter and assumes values between 0 and 1. Again, the memory function can be simplified in a Prony series of simple exponentials. From a physical perspective, it is assumed that the relaxation process can be divided into *N_m_* relaxation processes with different relaxation times and weight factors.

(21) $$M\left(\xi \right)=\sum_{i=1}^{N_{m}}\alpha_{i}^{M}\;exp(-\frac{\xi }{\tau_{i}^{M}})\;$$

(22) $$\sum_{i=1}^{N_{m}}\alpha_{i}^{M}\;=1\;$$

Each relaxation process is characterized by its fictive temperature:

(23) $$\frac{dT_{f_{i}}}{dt}=-\frac{T_{f_{i}}-T}{{\tau_{i}}^{M}}\frac{d\xi }{dt}$$

The weighted sum of partial fictive temperature is the actual fictive temperature.

For the resolution of previous equations, the implicit and unconditionally stable numerical algorithm proposed by Markovsky and Soules [35] was used. The partial fictive temperature at the timestep *k*, $T_{fi}(k)$, can be evaluated as the following:

(24) $$T_{f_{i}}\left(k\right)=\frac{{{\tau_{i}}^{M}\;T}_{f_{i}}\left(k-1\right)+T\left(k\right)dt\;a(T_{f}(k-1))}{{\tau_{i}}^{M}+dt\;a(T_{f}(k-1))}$$

(25) $$T_{f}\left(k\right)=\sum_{i=1}^{N}\alpha_{i}^{M}\;T_{f_{i}}\left(k\right)\;$$

As the expression proposed for the shift factor is not implemented in Ansys, the shift factor was given through a fictitious Arrhenius equation. The equality between Equation (14) and the Arrhenius expression was imposed:

(26) $$exp\left(\frac{\Delta H\left(\alpha \right)}{R}\left(\frac{x(\alpha )}{T}+\frac{1-x(\alpha )}{T_{f}}-\frac{1}{T_{g}\left(\alpha \right)}\right)\right)=exp\left(\frac{{\Delta H}_{Arhenius}}{R}\left(\frac{1}{T}-\frac{1}{T_{g}\left(\alpha \right)}\right)\right)$$

The “fictitious activation energy”,${\;\Delta H}_{Arhenius\;}$, that assures the equality of the two expressions, can be written as the following:

(27) $${\Delta H}_{Arhenius}(\alpha ,T_{f},T)=\frac{\Delta H\left(\alpha \right)\;T\;\left(T_{f}+T_{r}\left(-1+x\right)\right)-T_{g}(\alpha )\;x)}{T_{f}\;(T-T_{g}(a))}$$

The equivalent shift factor can be evaluated at each timestep based on the degree of conversion, temperature, and fictive temperature previously calculated.

The free strain *ε_free_* in Equation (6) for the case under study is the sum of the three components: the thermal strain due to temperature variations, the chemical strain due to the crosslinking reaction, and the strain due to the change in glass structure caused by structural relaxation. The incremental free strain for each timestep can be evaluated as the following:

(28) $$\Delta \varepsilon_{free}^{k}={{CTE}_{glass}}^{k}\;\left(T^{k}-T^{k-1}\right)+{{CTE}_{liquid}}^{k}\left({T_{f}}^{k}-{T_{f}}^{k-1}\right)-(\alpha^{k}-\alpha^{k-1})\lambda_{max}$$

The coefficient of thermal expansion is a linear function of the degree of conversion, and it assumes a different value in the glass or liquid state [36]. $\lambda_{max}$ is the maximum chemical contraction due to the crosslinking network formation.

### 2.2. Model Parameters

This article analyzes the physical behavior of 3501-6 epoxy resin (Hercules, Inc., Wilmington, DE, USA). It is a high-Tg commercial epoxy used mainly for aeronautical applications. The detailed chemical structure is proprietary; however, the resin is known to be a glycidyl ether of bisphenol A (DGEBA)-type resin cured with a multifunctional amine [21]. Because of its importance in the aeronautical sector, many researchers have experimentally characterized this kind of epoxy, and experimental data are available in the literature regarding curing kinetics and viscoelasticity.

In Table 2 are summarized the model parameters to describe each of involved physical phenomena.

Curing parameters are taken from the work of Bogetti et al. [28] and are reported in Table 1.

**Figure 2:**
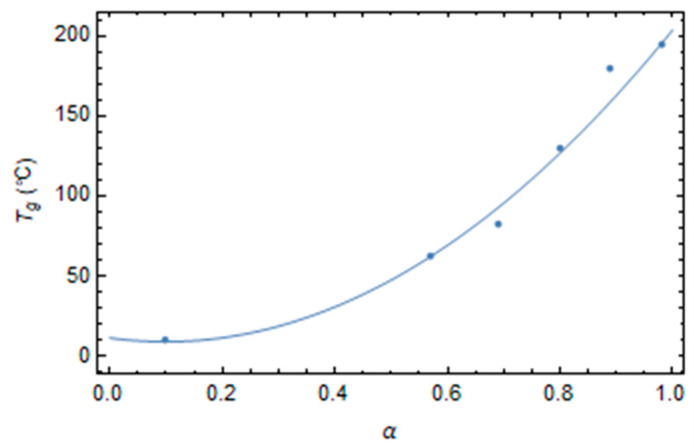
Glass transition temperature versus the degree of conversion.

Epoxy’s relaxation times are taken from the work of Kim and White [21], and the experimental values of *T_g_* are obtained from shift factors data, assuming that the fully cured epoxy’s glass transition temperature is 195 °C, and at the glass transition temperature, epoxy has the same relaxation time for each degree of conversion. The glass transition temperature data as a function of the degree of conversion exhibit a quadratic dependence, as shown in Figure 2. According to the experimental data, the reference time is assumed to equal 1s.

(29) $$T_{g}\left(\alpha \right)=11.46-47.33\;\alpha +{239.4\;\alpha }^{2}\;\;{}^{\circ}C\;$$

In order to characterize the epoxy’s viscoelastic behavior, we need to assign the instantaneous values of two viscoelastic functions and the Prony’s coefficients for the shear relaxation referred to the reference state (that is assumed to be at the glass transition temperature for each degree of curing). For the instantaneous value, we assume that the elastic modulus is 3200 MPa and the Poisson ratio is 0.35 [21]

**Figure 3:**
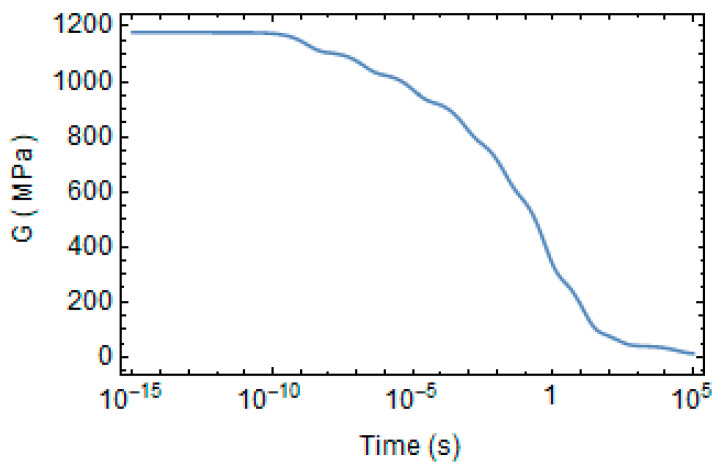
Evolution of shear relaxation modulus at the reference state.

The shear relaxation modulus at the reference state is plotted in Figure 3. It tends to vanish for a long time. Its Prony’s coefficients at the reference state are reported in Table 3.

The relaxation time in a generic temperature and degree of conversion’s state is the following:

(30) $$\tau \left(T,\alpha ,T_{f}\right)=\tau_{R}/a\left(T,\alpha ,T_{f}\right)$$

**Figure 4:**
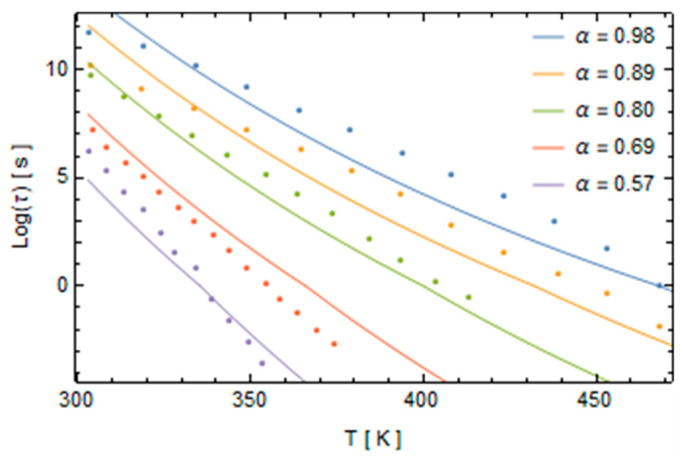
Relaxation time versus the temperature for various degrees of conversion.

The shift factor’s parameters are evaluated by fitting the experimental data by minimizing the interpolation error of Equation (30) with the data taken from the work of Kim and White and are reported in Figure 4.

The activation energy and the non-linearity parameter can be expressed as follows, respectively:

(31) $$\Delta H\left(\alpha \right)=8272-18541\;\alpha +2780\;\alpha^{2}$$

(32) $$x\left(\alpha \right)=0.833+0.0374\alpha \;$$

**Figure 5:**
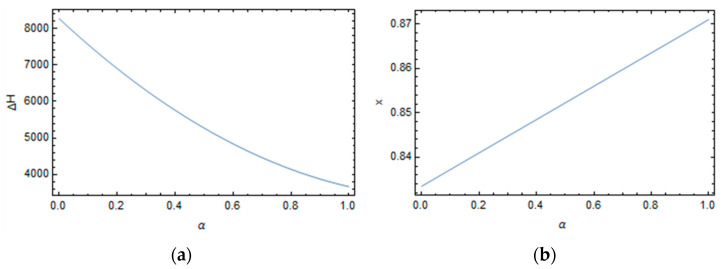
Shift factor’s parameters versus the degree of conversion. (**a**) Activation energy. (**b**) Non-linearity parameters.

The parameter *x* is almost constant with the degree of conversion. It suggests that independent of the degree of curing, the relative weights of structure and temperature on epoxy’s relaxation times are always the same. Conversely, the activation energy decreases with the degree of conversion, as reported in Figure 5.

The model, considering the extended ranges of temperatures and the degree of conversions, and the large number of involved phenomena, shows quite a good fitting of the experimental data as reported in Figure 4.

**Figure 6:**
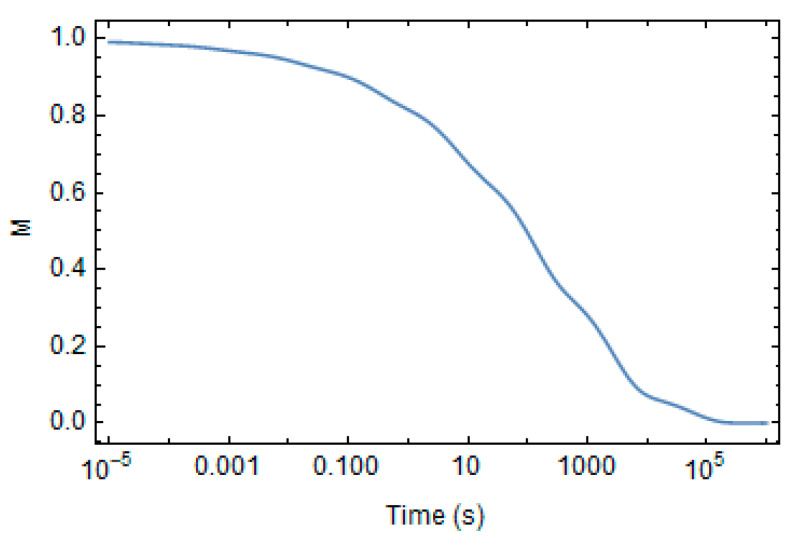
Memory function at the glass transition temperature.

Without experimental data for epoxy’s memory function, it is assumed that the epoxy behaves like another amorphous polymer [20] at the same distance from the respective glass transition temperatures. It is a plausible hypothesis because amorphous polymers at their glass transition temperature, measured at the same cooling condition, share similar characteristics regarding relaxation times and the broadness of the relaxation spectrum. The memory function at the reference state is plotted in Figure 6, and its Prony coefficients are reported in Table 4.

## 3. Results and Discussion

### 3.1. Effect of Curing on Structural Relaxation

**Figure 7:**
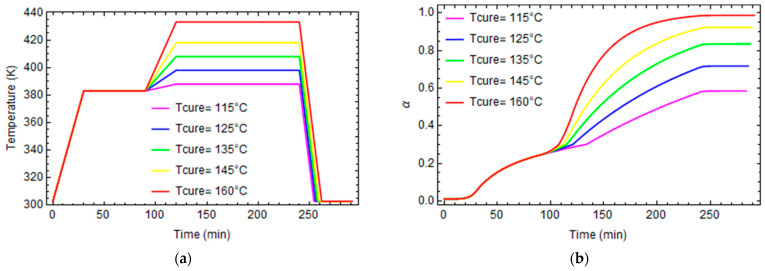
Temperature and degree of conversion profiles. (**a**) Temperature profiles. (**b**) Degree of conversion profiles.

In order to evaluate the proposed model’s reliability, the equations explained in the previous section are implemented in the software Mathematica 11.3, and the epoxy is virtually cured at five different temperatures varying between 115° C and 160 °C [21], to obtain at the end of curing five different degrees of conversion. Then, it is cooled to room temperature at a constant cooling rate of 0.1 C/sec, according to the thermal cycles described in Figure 7a. The evolution in time of the degree of conversion is plotted in Figure 7b.

The model can evaluate the fictive temperature’s evolution during the crosslinking reaction. Fictive temperatures are evaluated using the numerical algorithm explained in the previous section.

**Figure 8:**
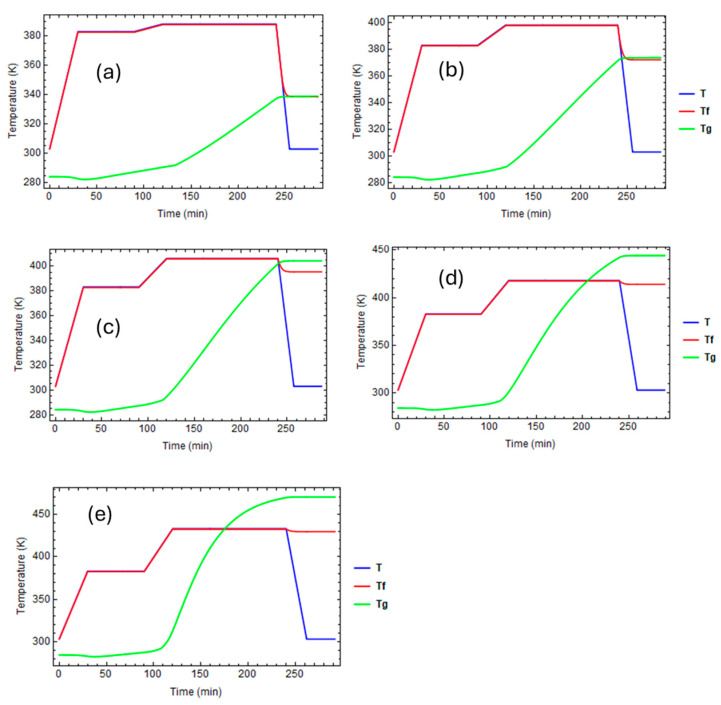
Evolution of temperature, fictive temperature, and glass transition temperature for different curing profiles: (**a**) Curing temperature Tcure = 115 °C. (**b**) Curing temperature Tcure = 125 °C. (**c**) Curing temperature Tcure = 135 °C. (**d**) Curing temperature Tcure = 145 °C. (**e**) Curing Temperature Tcure = 160 °C.

Figure 8 reports the evolution of temperatures, fictive temperatures, and glass transition temperatures for the various curing profiles.

In the first three cases (Figure 8a–c), the curing temperature is higher than the final glass transition temperature, so the epoxy is in its rubbery state until it cools to room temperature. When $T>T_{f}$, $T=T_{f}$, elsewhere, $T_{f}$ is almost equal to $T_{g}$.

In the last two cases (Figure 8d,e), the curing temperature is lower than the glass transition temperature at the end of the thermal cycle. So, the fictive temperature evolves until the glass transition temperature intersects the local temperature. At this time, the epoxy vitrifies, and its fictive temperature stops evolving.

The glass transition is a function of the cooling rate. Thus, samples at various degrees of conversion are cooled from T = 200 °C to 25 °C at different cooling rates ranging between 0.1 °C/s and 1000 °C/s.

**Figure 9:**
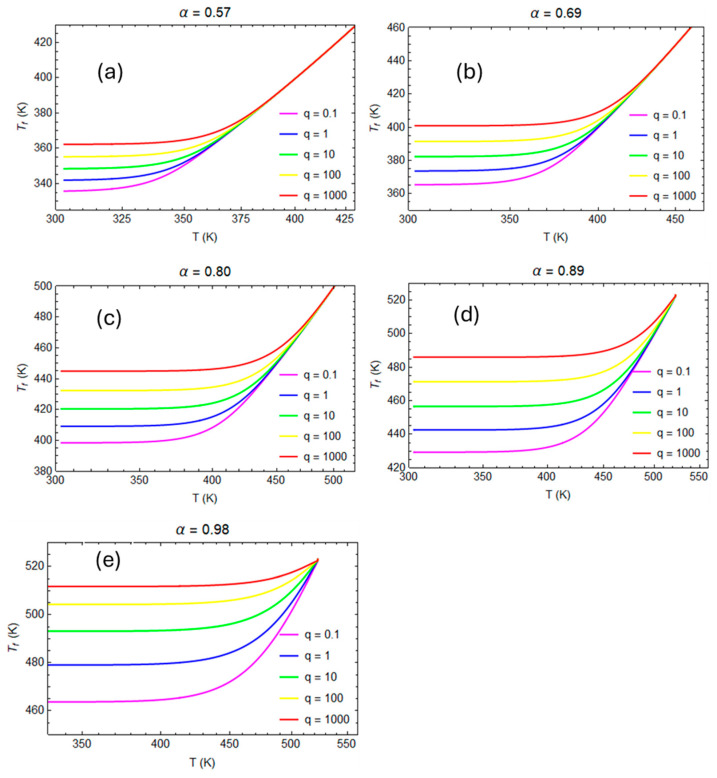
Evolution of fictive temperature during the cooling from liquid to glassy state for different cooling rates: (**a**) degree of conversion α = 0.57; (**b**) degree of conversion α = 0.69; (**c**) degree of conversion α = 0.80; (**d**) degree of conversion α = 0.89; (**e**) degree of conversion α = 0.98.

In Figure 9, the evolutions of the fictive temperature versus the local temperature during the cooling from a rubbery to a glassy state are reported for different conversion degrees.

When the cooling rate increases, the glass transition temperature increases, because the polymer’s structure has less time to evolve versus its equilibrium state. The actual glass transition temperature, *T_f_*’, is almost linear with the natural logarithm of the cooling rate.

**Figure 10:**
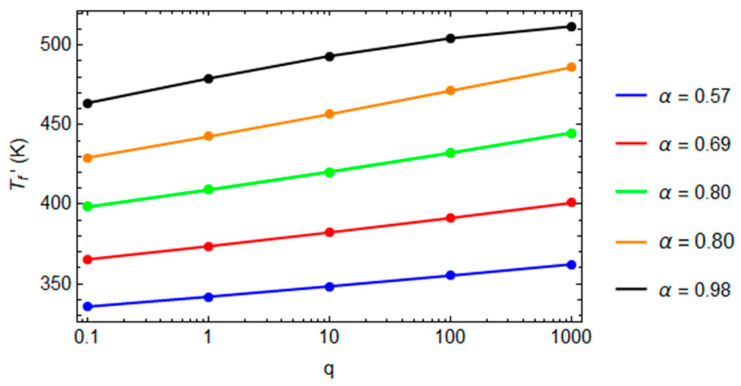
The fictive temperature at the end of cooling versus the logarithmic of the cooling rate.

For each degree of conversion, the slope of the curve in Figure 10 accords with Moynihan’s work [37]:

(33) $$\frac{d\;\ln q}{d\;T_{f}^{\prime }}=\frac{\Delta H(\alpha )}{R\;{T_{g}}^{2}\;}$$

### 3.2. Numerical Evaluation of Creep and Stress Relaxation

The coupons are cured according to curing cycles in Figure 7a, and after curing, they are numerically tested in creep and stress relaxation experiments using the software Ansys 18.0. The creep test applies a constant tensile force, and the strain evolution is monitored to evaluate the creep modulus.

(34) $$J=\frac{\varepsilon_{m}}{\sigma }\;$$

(35) $$\varepsilon_{m}=\frac{1}{V}\int \varepsilon \;dV\;$$

where *J* is the creep modulus, *σ* is the applied stress, and *ε_m_* is the mean strain measured on the coupon.

Similarly, a constant tensile displacement was applied over time in stress relaxation, and the evolution of stress was monitored to evaluate the relaxation modulus.

(36) $$\;E=\frac{\sigma_{m}}{\varepsilon \;\;}$$

(37) $$\sigma_{m}=\frac{1}{V}\int \sigma \;dV$$

where *E* is the relaxation modulus, *ε* is the applied strain, and *σ_m_* the mean stresses measured on the coupon.

**Figure 11:**
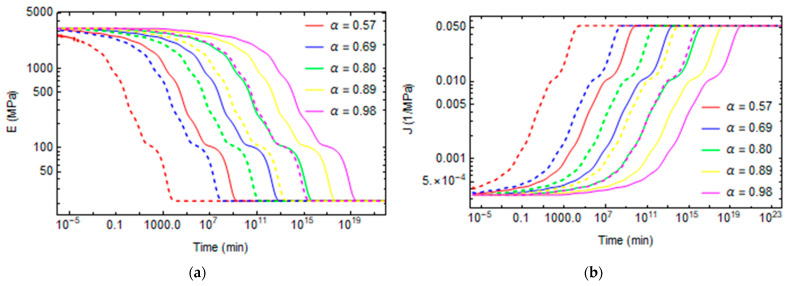
Stress relaxation and creep curves at T = 30 °C and T = 60 °C for different conversion degrees. (**a**) Stress relaxation (**b**) Creep. Continuing lines represent the relaxation phenomena at T = 30 °C. The dashed lines represent the relaxation phenomena at T = 60 °C.

**Figure 12:**
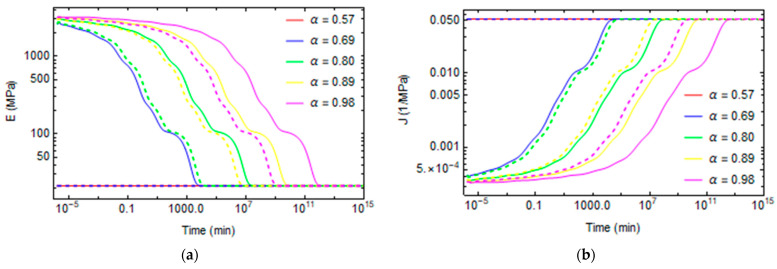
Stress relaxation and creep curves at T = 90 °C and T = 120 °C for different conversion degrees. (**a**) Stress relaxation (**b**) Creep. Continuing lines represent the relaxation phenomena at T = 90 °C. The dashed lines represent the relaxation phenomena at T = 120 °C.

Creep and stress relaxation tests are made at different conversion degrees between 0.57 and 0.98 and different temperatures varying from 30 °C to 120 °C. The stress relaxation tensile modulus for various degrees of conversions and temperatures are plotted in Figure 11a and Figure 12a. Similarly, the evolutions of creep compliance are shown in Figure 11b and Figure 12b.

**Figure 13:**
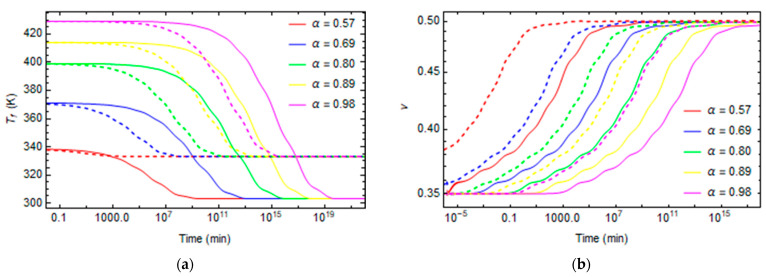
Fictive temperature and Poisson relaxation curves at T = 30°C and T = 60 °C for different conversion degrees. (**a**) Fictive temperature (**b**) Poisson. Continuing lines represent the relaxation phenomena at T = 30 °C. The dashed lines represent the relaxation phenomena at T = 60 °C.

**Figure 14:**
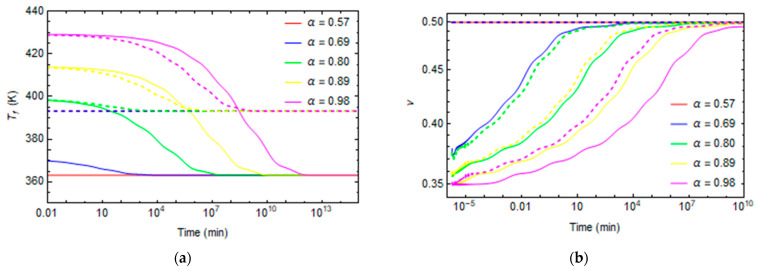
Fictive temperature and Poisson ratio evolution curves at T = 90 °C and T = 120 °C for different conversion degrees. (**a**) Fictive temperature (**b**) Poisson. Continuing lines represent the relaxation phenomena at T = 90 °C. The dashed lines represent the relaxation phenomena at T = 120 °C.

Figure 13a and Figure 14a plot the evolution of fictive temperature during aging at a constant temperature. The fictive temperature relaxes over time until it reaches the environment temperature. The relaxation is faster at a low degree of conversion because the polymer is less crosslinked, and the mobility of the structure is high. For the same reasons, relaxation phenomena occur more quickly at high temperatures.

The Poisson’s ratio evolves from 0.35, when the material demonstrates a solid-like behavior, until it reaches a 0.5 value at longer times, when the polymer’s behavior becomes rubber-like, as shown in Figure 13b and Figure 14b.

In some cases, at high temperatures and low degrees of conversions, at the beginning of the relaxation, the material is already in the rubbery state, so the relaxation properties remain constant at the rubbery values, as shown in Figure 12 and Figure 14 for the cases T = 90 °C and α = 0.57, T = 120 °C and α = 0.57, and T = 120 °C and α = 0.69.

## 4. Conclusions

This work proposed a numerical model to predict the effect of curing on commercial epoxy’s relaxation times. This model is based on a well-validated expression for the study of polymers’ glass transition and considers the effects of curing and structural relaxation, showing a good fitting of the experimental data.

This relaxation time expression was then implemented in an Ansys APDL environment and used as a shift factor to scale the viscoelastic properties as functions of the degree of conversion and temperature in order to simulate epoxy relaxation phenomena in terms of creep, stress relaxation, and fictive temperature evolution.

The results show that the temperature and degree of curing strongly affect the relaxation phenomena. For future perspectives, the proposed numerical results must be compared with experimental ones to check the proposed model’s reliability. In addition, in the next part of this work, the proposed model will be used to model the viscoelastic behavior of an epoxy component subjected to non-uniform thermal history to numerically predict the evolution of residual stresses in a polymeric part during its curing process.

## Figures and Tables

**Table 1:** Thermal and kinetic parameters for epoxy 3501.

A_1_	2.102 × 10^9^	min^−1^
A_2_	−2.014 × 10^9^	min^−1^
A_3_	1.960 × 10^5^	min^−1^
ΔE_1_	8.07 × 10^4^	J/mol
ΔE_2_	7.78 × 10^4^	J/mol
ΔE_3_	5.66 × 10^4^	J/mol
H_r_	473.16	kJ/Kg
R	8.314	J/Kg mol
ρ	1200	Kg/m^3^
c_p_	1260	J/Kg K
k	0.167	W/m K

**Table 2:** Model parameters.

Viscoelasticity	*G*_0_, *K*_0_, *τ_i_^G^*, *w_i_^G^* *i* = 1,…$n_{G}$
Structural Relaxation	*τ_i_^M^*, *α_i_^M^*, *T_f i_*, Δ*H*, *x* *i* = 1,…$n_{M}$
Curing	${\Delta E}_{i}$, *H_r_, A_i_* *i* = 1,…*3*
Glass Transition Temperature	*b*_1_, *b*_2_, *b*_3_

**Table 3:** Prony’s coefficients for shear relaxation module at the reference state.

N	τ_G_ (s)	α_i_^G^
1	1.75 × 10^−9^	0.059
2	1.75 × 10^−7^	0.066
3	1.09 × 10^−5^	0.083
4	6.60 × 10^−4^	0.112
5	1.70 × 10^−2^	0.154
6	4.76 × 10^−1^	0.262
7	1.17 × 10^1^	0.184
8	2.00 × 10^2^	0.049
9	2.95 × 10^4^	0.025

**Table 4:** Prony’s coefficients of memory function are at the reference state.

N	τ_M_ (s)	α_i_^M^
1	1.21 × 10^−6^	0.0062
2	2.60 × 10^−5^	0.0072
3	5.60 × 10^−4^	0.0175
4	1.21 × 10^−2^	0.0390
5	2.60 × 10^−1^	0.0856
6	5.60	0.1730
7	1.21 × 10^2^	0.2950
8	2.60 × 10^3^	0.298
9	5.60 × 10^4^	0.0785

## Data Availability

The raw data supporting the conclusions of this article will be made available by the authors on request.
